# Zinc as a Neuromodulator in the Central Nervous System with a Focus on the Olfactory Bulb

**DOI:** 10.3389/fncel.2017.00297

**Published:** 2017-09-21

**Authors:** Laura J. Blakemore, Paul Q. Trombley

**Affiliations:** ^1^Program in Neuroscience, Florida State University Tallahassee, FL, United States; ^2^Department of Biological Science, Florida State University Tallahassee, FL, United States

**Keywords:** zinc, olfactory bulb, central nervous system, modulation, amino acid receptors, voltage-gated ion channels

## Abstract

The olfactory bulb (OB) is central to the sense of smell, as it is the site of the first synaptic relay involved in the processing of odor information. Odor sensations are first transduced by olfactory sensory neurons (OSNs) before being transmitted, by way of the OB, to higher olfactory centers that mediate olfactory discrimination and perception. Zinc is a common trace element, and it is highly concentrated in the synaptic vesicles of subsets of glutamatergic neurons in some brain regions including the hippocampus and OB. In addition, zinc is contained in the synaptic vesicles of some glycinergic and GABAergic neurons. Thus, zinc released from synaptic vesicles is available to modulate synaptic transmission mediated by excitatory (e.g., N-methyl-D aspartate (NMDA), alpha-amino-3-hydroxy-5-methyl-4-isoxazolepropionic acid (AMPA)) and inhibitory (e.g., gamma-aminobutyric acid (GABA), glycine) amino acid receptors. Furthermore, extracellular zinc can alter the excitability of neurons through effects on a variety of voltage-gated ion channels. Consistent with the notion that zinc acts as a regulator of neuronal activity, we and others have shown zinc modulation (inhibition and/or potentiation) of amino acid receptors and voltage-gated ion channels expressed by OB neurons. This review summarizes the locations and release of vesicular zinc in the central nervous system (CNS), including in the OB. It also summarizes the effects of zinc on various amino acid receptors and ion channels involved in regulating synaptic transmission and neuronal excitability, with a special emphasis on the actions of zinc as a neuromodulator in the OB. An understanding of how neuroactive substances such as zinc modulate receptors and ion channels expressed by OB neurons will increase our understanding of the roles that synaptic circuits in the OB play in odor information processing and transmission.

## Introduction

Zinc is a common trace element that is found in multiple brain regions including the hippocampus, cerebral neocortex, hypothalamus, amygdala and olfactory bulb (OB; Donaldson et al., 1973; Gulya et al., 1991; Ono and Cherian, 1999). In addition to serving structural, catalytic, and regulatory functions, zinc is thought to play a role as a neuromodulator (Bitanihirwe and Cunningham, 2009; Nakashima and Dyck, 2009; Paoletti et al., 2009). This review summarizes the locations and release of vesicular zinc in the central nervous system (CNS), including in the OB. It also summarizes the effects of zinc on various receptors and ion channels involved in regulating neuronal excitability and synaptic transmission, with special emphasis on the actions of zinc as a neuromodulator in the OB.

Whereas most (80%–95%) zinc in the brain is tightly bound to proteins (Nakashima and Dyck, 2009; Paoletti et al., 2009), pools of chelatable zinc have been visualized with histochemical techniques and fluorescent probes (Danscher, 1981, 1996; Frederickson et al., 1983, 2000, 2005; Pérez-Clausell and Danscher, 1985; Frederickson, 1989; Blakemore et al., 2013). Although this chelatable zinc includes loosely bound zinc and ionic zinc, it primarily consists of vesicular zinc (Frederickson et al., 1983; Danscher et al., 1985; Pérez-Clausell and Danscher, 1985). A portion of zinc in the brain (15%) is contained in synaptic vesicles (Frederickson, 1989), with especially high concentrations found in the hippocampus (Danscher et al., 1985; Pérez-Clausell and Danscher, 1985; Slomianka et al., 1990) and OB (Friedman and Price, 1984; Masters et al., 1994; Jo et al., 2000; Blakemore et al., 2013). Most vesicular zinc is co-localized with glutamate in a subset of glutamatergic zinc-enriched neurons (Haug, 1967; Crawford and Connor, 1973; Frederickson et al., 1983; Pérez-Clausell and Danscher, 1985; Frederickson, 1989; Beaulieu et al., 1992). However, zinc is also contained in the synaptic vesicles of subpopulations of glycinergic and GABAergic neurons (Birinyi et al., 2001; Wang et al., 2001, 2002).

The co-localization of zinc and glutamate in synaptic vesicles, along with evidence that depolarizing stimuli release vesicular zinc (Assaf and Chung, 1984; Howell et al., 1984; Li et al., 2001; Blakemore et al., 2013), supports the view that neuronal activity can release zinc. Results from a variety of studies suggest that zinc modulates excitatory and inhibitory amino acid receptors and synaptic transmission (Smart et al., 1994; Bitanihirwe and Cunningham, 2009; Nakashima and Dyck, 2009; Paoletti et al., 2009). For example, zinc has been shown to inhibit gamma-aminobutyric acid (GABA) receptor-mediated and N-methyl-D aspartate (NMDA) receptor-mediated responses in the hippocampus (Westbrook and Mayer, 1987; Mayer and Vyklicky, 1989; Legendre and Westbrook, 1991; Xie et al., 1993; Smart et al., 1994). We have reported similar inhibition of NMDA- and GABA-receptor-mediated responses by zinc in OB neurons (Trombley and Shepherd, 1996; Trombley et al., 1998; Horning and Trombley, 2001; See Supplementary Table S1).

Various studies have explored zinc’s effects on other types of receptors and ion channels in the CNS, including metabotropic glutamate receptors, AMPA receptors (AMPARs), kainate receptors, glycine receptors, dopamine receptors, serotonin receptors, acetylcholine receptors, and P2X receptors as well as voltage-gated ion channels for Na^+^, K^+^, Ca^2+^, and Cl^−^ (Smart et al., 1994; Frederickson et al., 2005; Bitanihirwe and Cunningham, 2009; Nakashima and Dyck, 2009). In the OB, we (Horning and Trombley, 2001) and others (Puopolo and Belluzzi, 1998) have shown that zinc modulates several types of voltage-gated ion channels (see Supplementary Table S2). We have also demonstrated that the zinc has biphasic (potentiating/inhibiting) concentration-dependent effects on subsets of glycine receptors (Trombley and Shepherd, 1996; Trombley et al., 2011) and AMPARs (Blakemore and Trombley, 2004) in the OB (See Supplementary Table S1).

Sensory transduction of odor molecules occurs in olfactory sensory neurons (OSNs) and this information is transmitted to cortical structures that encode olfactory discrimination and perception (Trombley and Shepherd, 1993; Ennis et al., 2007, 2015). The first synaptic relay occurs in the OB, whose organization is critical to the processing of odor information. Excitatory and inhibitory amino acid receptors are widely distributed in the OB, with distinct laminar and cellular distributions (Trombley and Shepherd, 1993; Ennis et al., 2007). An understanding of how a neuroactive substance such as zinc modulates the multiple amino acid receptors and ion channels expressed by OB neurons will increase our understanding of the roles that synaptic circuits of OB play in odor information processing and transmission.

## Anatomy and Circuitry of The Olfactory Bulb

Odor molecules bind to receptors located on the cilia of OSNs in the olfactory epithelium. Odor binding activates cyclic nucleotide gated channels and the resulting depolarization evokes action potentials that are conducted along the olfactory nerve (ON) to the glomerular layer (GL) of the OB. The OB is divided into multiple layers, consisting of morphologically and functionally distinct types of cells (Ennis et al., 2007, 2015; Nagayama et al., 2014; Kosaka and Kosaka, 2016). See Figure 1 in Corthell et al. (2013) for a diagram illustrating the neuronal populations, layers and synaptic connections in the OB.

The GL is deep to the ON layer (made up of axons of OSNs) and it contains discreet spherical structures known as glomeruli (Golgi, 1875). Within glomeruli, axons of OSNs form glutamatergic axodendritic synapses with mitral and tufted (M/T) cells (Berkowicz et al., 1994; Ennis et al., 1996) as well as intrinsic interneurons (Ennis et al., 2001). M/T cells represent the major output cells in the OB, and these cells are also glutamatergic (Liu et al., 1989; Trombley and Westbrook, 1990; Christie et al., 2001). Three morphologically distinct populations of OB interneurons surround the glomeruli, known collectively as juxtaglomerular (JG) cells: periglomerular (PG) cells, short axon (SA) cells and external tufted (ET) cells (Golgi, 1875; Pinching and Powell, 1971; Shepherd, 1972). JG cells make synaptic contacts with each other as well as with OSN terminals and M/T cells (Shepherd, 1972; Hsia et al., 1999; Berkowicz and Trombley, 2000; Ennis et al., 2001, 2015; Davila et al., 2003; Nagayama et al., 2014; Kosaka and Kosaka, 2016; Liu et al., 2016; Vaaga et al., 2017).

Within the glomeruli, several types of circuits exist (Ennis et al., 2015). M/T cells provide excitatory (glutamatergic) input to PG cells, which provide feedback and feedforward inhibitory input onto M/T cells at reciprocal dendrodendritic GABAergic synapses (Shepherd et al., 2004; Ennis et al., 2007, 2015). This local intraglomerular inhibition is mostly mediated by GABA_A_ receptors (Ennis et al., 2007, 2015). More recent evidence suggests that ET cells play a role in this inhibition. Spontaneous and ON-evoked spike bursts from ET cells drive most PG cells, and this ET cell→PG cell circuit has been shown to result in a major part of the intraglomerular inhibition feeding forward onto M/T cells and feeding back onto ET cells (Ennis et al., 2015). It also has been shown that OSN terminals synapse on ET cells, which, in turn, provide glutamatergic excitatory input to M/T cells and all other JG cells (Hayar et al., 2004a,b, 2005; De Saint Jan et al., 2009; Gire et al., 2012). These findings suggest that ON→M/T cell and ON→ET cell→M/T cell circuits operate in parallel to generate glomerular output (De Saint Jan et al., 2009; Gire et al., 2012; Ennis et al., 2015).

Spanning multiple glomeruli are the dendrites and axons of SA cells, which are thought to regulate interglomerular functions (Aungst et al., 2003; Ennis et al., 2007). Recently, it was shown that GABA and dopamine are co-released from a subset of SA cells, which evokes a temporally biphasic inhibition-excitation response in ET cells (Liu et al., 2013) and reduces OSN glutamate release probability via D_2_ and GABA_B_ receptor activation (Vaaga et al., 2017). Some data suggest that SA cells also may inhibit mitral cells via actions at GABA_A_ receptors (Liu et al., 2016).

Deep to the GL layer is the external plexiform layer (EPL), which mostly consists of mitral cell and granule cell dendrites that come from the mitral cell layer (MCL) and granule cell layer (GCL), respectively. The EPL also includes the cell bodies and dendrites of tufted cells and heterogeneous subtypes of intrinsic interneurons (Ennis et al., 2015). The EPL contains dendrodendritic synapses, which are reciprocal excitatory/inhibitory synapses between M/T cell and granule cell dendrites (Hirata, 1964; Rall et al., 1966; Price and Powell, 1970b; Woolf et al., 1991). Such synapses provide feedback and lateral inhibition of M/T cells (Ennis et al., 2015). Glutamate released from M/T cells evokes a dual-component response in granule cells, consisting of fast AMPAR-mediated and slower NMDA-receptor-mediated responses (Trombley and Westbrook, 1990; Isaacson and Strowbridge, 1998; Schoppa et al., 1998; Aroniadou-Anderjaska et al., 1999; Chen et al., 2000; Isaacson, 2001). Such activation of granule cells induces GABA_A_ receptor-mediated inhibition of M/T cells (Chen et al., 2000; Isaacson and Vitten, 2003; Dietz and Murthy, 2005). GABA release is coupled to calcium influx via both NMDA receptors (NMDARs) and voltage gated P/Q- and N-type calcium channels (Isaacson and Strowbridge, 1998; Chen et al., 2000; Halabisky et al., 2000; Isaacson, 2001; Egger et al., 2003, 2005).

Below the EPL is the MCL, which contains the somata of mitral cells and granule cells (Ennis et al., 2015). Axon collaterals of mitral cells terminate in the internal plexiform layer (IPL) and GCL (Price and Powell, 1970a; Mori et al., 1983) and also exit the OB as the lateral olfactory tract, which projects to the primary olfactory cortex and other olfactory encoding structures (de Olmos et al., 1978; Ennis et al., 2007, 2015).

Below the MCL is the IPL, which is composed mostly of dendrites of granule cells and axons of M/T cells and centrifugal sources (Ennis et al., 2007). Deep to the IPL is the GCL, which is occupied mostly by granule cells (Nagayama et al., 2014). Granule cells are axon-less inhibitory GABAergic cells whose dendritic spines form reciprocal synapses with secondary dendrites of M/T cells in the EPL (Nagayama et al., 2014). The IPL and GCL also contain a group of diverse interneurons loosely referred to as deep SA cells that include horizontal, Blanes, Golgi and Cajal cells (Eyre et al., 2008).

## Zinc Transporters

As well as modulating synaptic transmission, zinc functions as an intracellular signal transducer, which is regulated by zinc transporters (Hara et al., 2017). Zinc transporters can be categorized into two major families: SLC39s/ZIPs and SLC30s/ZnTs (Hojyo and Fukada, 2016; Hara et al., 2017). The ZIP family is responsible for zinc uptake into the cells (Fukada et al., 2014; Hojyo and Fukada, 2016), while the ZnT family reduces the intracellular cytoplasmic zinc content by effluxing zinc from the cytosol or transporting it into vesicles or intracellular organelles (Fukada et al., 2014; Hojyo and Fukada, 2016).

Several studies described here have examined the distribution and/or function of ZnTs. ZnT-1 protects cells against zinc toxicity (Shusterman et al., 2014) and regulates cellular zinc levels (Segal et al., 2004). ZnT-3 is responsible for zinc uptake into synaptic vesicles (Palmiter et al., 1996; Cole et al., 1999), thus, has served as another marker of zinc-enriched terminals. The pattern of ZnT3 mRNA expression in the brain is closely related to the distribution of zinc-containing neurons (McAllister and Dyck, 2017) and most regions of the brains of ZnT3 knockout (KO) mice lack free histochemically reactive zinc (Cole et al., 1999; McAllister and Dyck, 2017).

## Locations of Vesicular Zinc

Vesicular zinc is most often contained in subpopulations of glutamatergic neurons (Beaulieu et al., 1992; Slomianka, 1992). Some vesicular zinc is also found in glycinergic and GABAergic neurons in the spinal cord and cerebellum (Birinyi et al., 2001; Wang et al., 2001, 2002). The hippocampus, amygdala, cortex and OB are rich in zinc-containing glutamatergic neurons (Dyck et al., 1993; Frederickson and Moncrieff, 1994; Pérez-Clausell, 1996; Ichinohe and Rockland, 2005). These neurons project internally to sites within the telencephalon (Nakashima and Dyck, 2009), with their pathways mostly found in the cerebral cortex and limbic regions of the forebrain (Bitanihirwe and Cunningham, 2009).

Zinc in the OB is highly concentrated in the GL and GCL (Friedman and Price, 1984; Jo et al., 2000; Sekler et al., 2002; Blakemore et al., 2013). Early histochemical studies demonstrated high concentrations of zinc in the vesicular compartment of the OB (Friedman and Price, 1984; Pérez-Clausell and Danscher, 1985; Masters et al., 1994). In a 2000 study, zinc autometallography (AMG) and ZnT3 immunocytochemistry showed zinc-enriched presynaptic terminals that make contact with granule cells in the GCL and M/T cells and PG cells in the GL (Jo et al., 2000). They suggested that the two sources of zinc-enriched synapses in the mouse OB are centrifugal fibers projecting to granule cells and PG cells and OSN terminals contacting dendrites of M/T cells and PG cells (see Figure 6 in Jo et al., 2000 for a schematic diagram of potential zinc-enriched terminals projecting to the OB). These investigators later used zinc selenium AMG to examine the origins of centrifugal zinc-enriched pathways that project to the rat OB (Mook Jo et al., 2002). They found that the main centrifugal sources of zinc-enriched terminals in the rat OB originate from the nuclei composing the primary olfactory cortex. Collectively, these results suggest that olfactory zinc-enriched terminals are involved in odor information processing.

Another study explored the complementary distributions of chelatable zinc and the zinc transporter ZnT-1 in the mouse OB (Sekler et al., 2002). Zinc, visualized with TSQ histofluorescence, was most heavily concentrated in the olfactory glomeruli, the IPL, and the GCL; negligible zinc was seen in the EPL and MCL. ZnT-1 immunoreactivity was most pronounced in the MCL and in PG cells surrounding the glomeruli, with moderate labeling of granule cells. In brain sections, ZnT-1- immunoreactive neurons in the OB were closely related to synaptic zinc.

As previous studies only characterized zinc’s location in the OB in fixed tissues, we recently conducted a study aimed at advancing these observations by using the zinc-sensitive, fluorescent probe Zinpyr-1 (ZP1; Burdette et al., 2001) to investigate the location and release of zinc in living OB slices (Blakemore et al., 2013). ZP1 is a membrane-permeable probe with a fluorescence turn-on that is selective for ionic zinc, thus, permitting zinc in synaptic vesicles to be viewed (Blakemore et al., 2013).

First, we used ZP1 to visualize the location of vesicular zinc in OB slices from 19 to 21-day-old rats (Blakemore et al., 2013). We found high-intensity ZP1 fluorescence in both the GL (vesicular zinc from OSN terminals and centrifugal fibers) and the GCL (vesicular zinc from centrifugal fibers); only a very weak signal was seen in the EPL, which does not contain much zinc.

To demonstrate that the observed signals reflect free ionic zinc, we applied the zinc chelators tris(2-pyridylmethyl)amine (TPA, 30 μM; Ghosh et al., 2010; Huang et al., 2013) and *N,N,N′,N′*-tetrakis (2-pyridylmethyl)ethylenediamine (TPEN, 30 μM) to absorb zinc ions from ZP1-treated slices. Incubation with either chelator produced similar reductions in the ZP1 signal, suggesting that the fluorescence emission from ZP1 denotes binding to ionic zinc (Blakemore et al., 2013). Incubation of the ZP1-treated OB slices in a high (50 mM) extracellular potassium solution also rapidly eliminated the zinc signal, likely by causing the release of zinc-containing synaptic vesicles due to depolarization. Together, these results obtained with the ZP1 probe and zinc chelators demonstrated, for the first time in living OB slices, that vesicular zinc is mostly localized within the GL and GCL.

## Release of Vesicular Zinc

The co-localization of zinc and glutamate in a subset of zinc-enriched neurons, along with evidence that zinc is released from synaptic vesicles in response to depolarizing stimuli, suggests that zinc is released during synaptic transmission. In early (1980s) experiments, the release of zinc into the synaptic cleft was first demonstrated *in vitro* in hippocampal slice preparations following depolarization in response to electrical (Howell et al., 1984) or chemical (high potassium or kainate; Assaf and Chung, 1984) stimuli. This zinc release was both calcium- and depolarization-dependent (Assaf and Chung, 1984; Howell et al., 1984), consistent with a vesicular release mechanism.

Various tools for studying vesicular zinc have recently emerged. These include highly selective fluorescent probes that can be used to detect mobile zinc in live cells and tissues, different types of KO mice (e.g., ZnT3 KO mice lacking vesicular zinc; Palmiter et al., 1996; Cole et al., 1999), and various zinc chelators (Goldberg et al., 2016).

Zinc imaging combined with electrophysiological recordings has provided additional direct evidence of vesicular zinc release (Vogt et al., 2000; Li et al., 2001; Molnár and Nadler, 2001; Ueno et al., 2002; Qian and Noebels, 2005, 2006; Paoletti et al., 2009). Using microfluorescence imaging in rat hippocampal slices, Li et al. (2001) showed that electrical stimulation of mossy fibers (MFs) caused the immediate release of zinc from synaptic terminals into the extracellular microenvironment. The membrane-impermeable, zinc-selective fluorescent dye FluoZin-3 (Gee et al., 2002) was used to demonstrate zinc release during exocytosis evoked by action potentials in hippocampal slices at zinc-enriched MF (Qian and Noebels, 2005) and CA3–CA1 (Qian and Noebels, 2006) synapses. The absence of zinc signal observed in slices from ZnT3 KO mice indicated that the released zinc came from synaptic vesicles (Qian and Noebels, 2005, 2006).

Another study in the rat hippocampus used the selective membrane-impermeable zinc chelator ZX1 (Burdette et al., 2001; Chang and Lippard, 2006; Zhang et al., 2007) to show that vesicular zinc released in response to electrophysiological stimulation modulates long-term potentiation (LTP) at MF-CA3 synapses (Pan et al., 2011). A subsequent study used genetic tools (e.g., KO and knockin mice), the fast chelating agent tricine (Paoletti et al., 1997), and electrophysiological recordings to demonstrate that vesicular zinc released via exocytosis modulates activity at postsynaptic NMDARs at the MF-CA3 and Schaffer collateral-CA1 synapses (Vergnano et al., 2014).

In a series of experiments performed in the zinc-enriched dorsal cochlear nucleus (DCN), the effects of both synaptic and tonic (nonsynaptic) zinc were explored. In one study, ZX1, electrophysiological methods, and glutamate uncaging were used to demonstrate that action potential-evoked release of synaptic zinc was required to inhibit alpha-amino-3-hydroxy-5-methyl-4-isoxazolepropionic acid (AMPA) receptor-mediated currents (Kalappa et al., 2015). In another study, chelation of zinc with ZX1 increased the frequency of action potentials in the DCN. This is consistent with their interpretation that tonic zinc inhibits spontaneous action potentials in the DCN by a combination of glycine receptor potentiation and an increase in glycine release probability (Perez-Rosello et al., 2015). Anderson et al. (2015) also used ZX1 and a novel extracelluar ratiometric fluorescent zinc sensor (LZ9) to show that both synaptic and tonic zinc modulate extrasynaptic NMDARs in the DCN. Finally, Perez-Rosello et al. (2013) found that synaptic zinc in the DCN reduces the probability of transmitter (glutamate) release by acting with the putative metabotropic zinc-sensing receptor GPR39 to promote endocannabinoid synthesis.

Recently, we used zinc imaging combined with electrophysiology to explore the release of synaptic zinc in the OB (Blakemore et al., 2013). We incubated OB slices from 19 to 21-day-old rats with ZP1 and induced zinc release by electrically stimulating the ON layer. Placement of the stimulating electrode was at the ON-GL boundary to permit isolation of a single or several glomeruli innervated by the adjacent bundle of ON axons. The ON axon bundle was electrically stimulated with a pattern designed to mimic the breathing cycle in rats (Youngentob et al., 1987). These stimulation patterns selectively decreased the zinc signal in glomeruli adjacent to the stimulating electrode, whereas the zinc signal in nearby glomeruli remained unchanged. Thus, we have presented the first evidence that OSN terminals within individual glomeruli release vesicular zinc in response to patterned electrical stimulation of the ON simulating the rat’s breathing cycle. This observation is important given that the GL expresses a high density of neurotransmitter receptor and ion channel targets for modulation by zinc co-released with glutamate from OSNs.

The exact quantity of vesicular zinc released into the synaptic cleft is unclear. The concentration of zinc in the synaptic cleft after an excitatory event is influenced by factors such as the number of zinc vesicles that fuse to the presynaptic membrane (Goldberg et al., 2016). Early studies suggested that extracellular zinc concentrations ranged from 10 to 300 μM following depolarization of neurons (Frederickson et al., 1983; Assaf and Chung, 1984; Howell et al., 1984; Xie and Smart, 1991). However, synaptic concentrations of zinc could be greater given that the synaptic cleft comprises only part of the extracellular space. More recently, it has been estimated that peak zinc concentrations in the synaptic cleft following exocytosis range from 10 nM to >100 μM (Vogt et al., 2000; Ueno et al., 2002; Paoletti et al., 2009; Vergnano et al., 2014; Goldberg et al., 2016). Synaptic concentrations of zinc in the micromolar range are not unexpected since the synaptic concentrations of glutamate (Clements et al., 1992), GABA (Jones and Westbrook, 1995), and glycine (Beato, 2008) all exceed 1 mM. This supports the notion that vesicular release of zinc generates synaptic concentrations well within a range to effectively alter ion channel function.

## Zinc Modulation of Amino Acid Receptors and Synaptic Transmission with Focus on the OB

A number of studies suggest that zinc modulates amino acid receptors and synaptic transmission (Smart et al., 1994; Bitanihirwe and Cunningham, 2009; Nakashima and Dyck, 2009; Paoletti et al., 2009). This section examines the effects of zinc on several types of excitatory amino acid receptors (NMDARs, AMPARs) and inhibitory amino acid receptors (GABA_A_ receptors, glycine receptors), with special emphasis on zinc’s actions in the OB.

### Zinc’s Effects on NMDA Receptors

Much attention has been focused on the effects of zinc on NMDARs given their role in neuronal excitability and plasticity (Nakashima and Dyck, 2009). Early studies showed potent inhibition of NMDARs by zinc at a range of zinc concentrations (1 μM–1 mM; Smart et al., 1994). At low micromolar concentrations, zinc caused voltage-independent non-competitive inhibition of NMDAR-mediated responses in hippocampal and cortical neurons by decreasing open channel probability (Peters et al., 1987; Westbrook and Mayer, 1987; Mayer et al., 1989; Legendre and Westbrook, 1990; Xie et al., 1993; Paoletti et al., 2009). At higher concentrations, zinc bound to the magnesium site within the channel causing voltage-dependent inhibition of NMDAR-mediated currents (Paoletti et al., 2009).

NMDARs are formed by multiple subunits and zinc’s effects on NMDARs vary with subunit composition. In 2009, new nomenclature to describe these subunits was introduced. The seven different subunits described consist of the GluN1 subunit, four different GluN2 subunits (GluN2A, GluN2B, GluN2C and GluN2D), and two GluN3 subunits (GluN3A and GluN3B; see Table 3 in Collingridge et al., 2009). Here, to avoid confusion, we use the nomenclature used in previously published articles with the new nomenclature indicated parenthetically.

Whereas zinc is a specific antagonist of some GluN2-containing NMDARs (Paoletti et al., 2013), differences in zinc affinity for various GluN2 subunits exist. Zinc causes a high affinity voltage-independent inhibition of NR1/NR2A (GluN1/GluN2A)-containing receptors, and a low affinity inhibition of NR1/NR2B (GluN1/GluN2B)-containing receptors (Chen et al., 1997; Paoletti et al., 1997; Low et al., 2000; Rachline et al., 2005). The functional consequence of this difference in zinc affinity is that low (nanomolar) zinc concentrations only inhibit GluN2A-containing receptors, whereas higher (micromolar) zinc concentrations are needed to inhibit GluN2B-containing receptors (Paoletti et al., 1997, 2013; Rachline et al., 2005).

Early autoradiographic, immunocytochemical, *in situ* hybridization and electrophysiological studies demonstrated the presence of NMDARs in the OB (Trombley and Shepherd, 1993). NMDARs are found throughout the OB aside from the IPL (Watanabe et al., 1993; Monyer et al., 1994; Petralia et al., 1994; Ennis et al., 2007).

We used whole-cell recording techniques to examine the effects of zinc on NMDARs expressed by OB neurons. We found that zinc was an effective antagonist of NMDA-mediated currents in rat OB neurons in primary culture and acutely isolated from adult animals (Trombley and Shepherd, 1996). Whereas 1 μM zinc had little effect on currents evoked by 100 μM NMDA, 100 μM zinc almost completed blocked these currents. The half maximal inhibitory concentration (IC50) was 19 μM (Trombley and Shepherd, 1996).

In our 1998 study, we investigated zinc’s effect on spontaneous, glutamate-mediated excitatory activity in the rat OB, mediated in part by NMDARs (as well as AMPA and kainate receptors; Trombley et al., 1998). At 100 μM, zinc caused a transition from asynchronous spiking to synchronous busting while also reducing synaptic frequency. Our subsequent study showed similar inhibitory effects of zinc (100 μM) on spontaneous excitatory activity (Horning and Trombley, 2001).

Because glutamate and zinc are co-localized in OSN terminals, there could be a variety of outcomes in regard to modulation of odor processing depending on their patterns of synaptic release. Both NMDA and AMPARs are involved in the activation of OB neurons by OSNs (Berkowicz et al., 1994; Ennis et al., 1996, 2001; Aroniadou-Anderjaska et al., 1997; Chen and Shepherd, 1997). Thus, inhibition of NMDARs by zinc co-released with glutamate from OSN terminals may modify excitatory transmission at these synapses. The NMDAR-mediated component of the response of M/T cells to glutamate released from OSNs is unusually long, resulting in a late spiking component (Ennis et al., 1996, 2001; Aroniadou-Anderjaska et al., 1997). Zinc may attenuate this NMDAR-mediated component of the response to ON input, thus, alter the spiking rate.

### Zinc’s Effects on AMPARs

The AMPA-subtype of glutamate receptors mediates most fast excitatory transmission in the CNS. AMPARs play an important role in normal function and plasticity of the brain. Zinc often had biphasic, concentration-dependent effects at AMPARs expressed in Xenopus oocytes (Rassendren et al., 1990) or on neurons from various brain regions (Mayer et al., 1989; Bresink et al., 1996). Low concentrations of zinc (50–300 μM) have been shown to potentiate AMPAR-mediated currents, while higher concentrations (1–3 mM) inhibit these currents (Mayer et al., 1989; Bresink et al., 1996). Furthermore, zinc-sensitive and zinc-insensitive AMPARs have been shown to co-exist in some brain regions (Mayer et al., 1989; Lin et al., 2001).

Early autoradiographic, immunocytochemical, *in situ* hybridization, and electrophysiological studies demonstrated the presence of AMPARs in the OB (Trombley and Shepherd, 1993). Apart from the ONL and subependymal layer, AMPARs are distributed throughout the OB (Petralia and Wenthold, 1992; Martin et al., 1993; Molnár et al., 1993; van den Pol, 1995; Ennis et al., 2007). AMPARs are found on the cell bodies and dendrites of mitral, tufted and JG cells (Petralia and Wenthold, 1992; Molnár et al., 1993; Giustetto et al., 1997; Montague and Greer, 1999). Because zinc and glutamate are co-localized in synaptic vesicles within the OB, we used whole-cell recording techniques to examine the effects of zinc on AMPARs on cultured rat OB neurons (Blakemore and Trombley, 2004). We found that the effects of various concentrations of zinc on AMPA (50 μM)-evoked currents mediated by OB AMPARs can be biphasic, uniphasic, or absent.

In contrast to previous studies in other brain regions, we analyzed the effects of multiple concentrations of zinc (30 μM, 100 μM and 1 mM) on AMPARs in the same cell in a subpopulation of OB neurons (*N* = 47; Blakemore and Trombley, 2004). We observed the expected biphasic response to zinc, current potentiation at 30 μM and/or 100 μM and inhibition at 1 mM, in ~46% of M/T cells compared with only ~16% interneurons. This difference in the frequency of a biphasic response between cell types was statistically significant. AMPARs on no M/T cells and two interneurons were insensitive to all three zinc concentrations. Uniphasic potentiating-only or inhibiting-only responses were observed at AMPARs on the remaining cells. Thus, the range of zinc concentrations we used was more likely to generate uniphasic rather than biphasic effects at AMPARs in the OB.

AMPARs are formed from four subunits: GluR1–4 (Dingledine et al., 1999). In 2009, new nomenclature to describe these subunits was introduced (see Table 3 in Collingridge et al., 2009), which re-named GluR1–4 as GluA1–4. Here, to avoid confusion, we use the nomenclature used in previously published articles with the new nomenclature indicated parenthetically. Zinc’s effects on AMPARs vary with the receptor’s subunit composition (Dreixler and Leonard, 1994), extracellular environment (Dreixler and Leonard, 1997) and alternative splicing of the AMPAR gene (Shen and Yang, 1999). In *Xenopus* oocytes, low concentrations of zinc (4–7.5 μM) augmented currents mediated by homomeric GluR3 (GluA3), but not homomeric GluR1 (GluA1) receptors, suggesting the GluR3 (GluA3) subunit is necessary for zinc potentiation (Dreixler and Leonard, 1994). Heteromeric expression of GluR3 (GluA3) with GluR2 (GluA2) and GluR1 (GluA1) with GluR2 (GluA2) both resulted in the absence of zinc potentiation (Dreixler and Leonard, 1994). Dreixler and Leonard later suggested that the zinc potentiation site in AMPARs may encompass more than one GluR3 (GluA3) subunit and that this site may be altered when the GluR2 (GluA2) subunit is included in the receptor (Dreixler and Leonard, 1997). The responsiveness of AMPARs to zinc is also affected by alternative splicing of the AMPAR gene, with resistance to modulation by zinc seen in flop variants but not flip variants (Shen and Yang, 1999).

Given these findings, one explanation for the differences in zinc sensitivity among OB neurons observed in our 2004 study is that AMPAR subunits are heterogeneously distributed within the OB. Support for this stems from immunohistochemical data showing that all AMPAR subunit proteins are expressed in the OB but follow distinct laminar, cellular and subcellular distributions (Montague and Greer, 1999). Our previous finding that the kinetics of OB AMPARs vary within and between OB neuron subtypes (Blakemore and Trombley, 2003) further suggests the diverse expression of AMPAR subunits and products of alternative splicing of the AMPAR gene (Dingledine et al., 1999).

In our 2003 study of AMPAR kinetics, we observed differences in the rate and extent of receptor desensitization between M/T cells and interneurons in primary culture (Blakemore and Trombley, 2003). We also observed differences between M/T cells and interneurons in their AMPARs’ sensitivity to cyclothiazide (Blakemore and Trombley, 2003), a drug that reduces desensitization of flip splice variants more extensively than flop splice variants (Dingledine et al., 1999). Our results suggest that AMPARs on interneurons contain a relatively higher percentage of flop subunits, whereas those on M/T cells contain a relatively higher percentage of flip subunits (Blakemore and Trombley, 2003). Given the results of Shen and Yang (1999), this may explain why AMPARs on OB interneurons were less likely than AMPARs on M/T cells to show a biphasic response to zinc (Blakemore and Trombley, 2004). It also has been reported that application of cyclothiazide abolishes zinc-mediated potentiation of currents evoked by glutamate, suggesting that zinc may potentiate AMPAR-receptor mediated currents by inhibiting receptor desensitization (Lin et al., 2001).

Evidence from our molecular biology study (Horning et al., 2004) provides support for the conclusions from our kinetic study that the OB has a diverse expression of AMPAR subunits and splice variants. In this study, we found that while the rat OB expresses mRNAs for GluR1–4 (GluA1–4), the relative amounts differ i.e., GluR2 > GluR1 >> GluR4 >> GluR3. Similar amounts of both variants were found in GluR2 and GluR4 transcripts (GluR2: ~55% flip, ~45% flop; GluR4: ~60% flip, ~40% flop). However, GluR1 and GluR3 transcripts consisted mostly of flip (GluR1: ~95% flip, ~5% flop; GluR3: ~93% flip, ~7% flop; Horning et al., 2004). The relatively higher levels of the flop variant for the GluA2 subunit may suggest that interneurons are more likely to contain GluA2, thus, are less likely to be sensitive to zinc.

Our 2004 study also showed that the frequency and magnitude of zinc’s effects at AMPARs on OB neurons vary with cell type. Modulation of AMPAR-mediated currents was observed in a greater percentage of M/T cells than interneurons at low (30 μM, 100 μM) but not high (1 mM) zinc concentrations. However, zinc’s effect on AMPAR-mediated currents was greater in magnitude in interneurons than in M/T cells (Blakemore and Trombley, 2004). AMPARs that lack (or contain unedited) GluR2 (GluA2) are also highly permeable to calcium (Hume et al., 1991; Verdoorn et al., 1991; Burnashev et al., 1995) and evidence suggests that OB interneurons primarily express AMPARs with low Ca^2+^ permeability (Jardemark et al., 1997). Thus, our observation that zinc modulated AMPARs on interneurons less often than AMPARs on M/T cells again may reflect the fact that AMPARs on interneurons have a higher GluA2 content.

In later studies, we (Blakemore et al., 2006) and others (Ma and Lowe, 2007; Pimentel and Margrie, 2008) demonstrated that a subset of AMPARs activated by OSNs, as well as AMPARs activated between M/T cell dendrites, are calcium permeable. This finding may be important, as calcium-permeable AMPA/kainate receptors on cortical and hippocampal neurons have been shown to also flux zinc, with a myriad of effects in the postsynaptic neuron (Yin and Weiss, 1995; Weiss and Sensi, 2000). Once within the cell, zinc can trigger widespread disruptions of normal cellular functions, including disruption of calcium homeostasis and tubulin assembly, inhibition of mitochondrial electron transport, over-activation of calcium-mediated enzymes, and the production of reactive oxygen species (Kress et al., 1981; Csermely et al., 1988; Choi and Koh, 1998; Weiss and Sensi, 2000). Zinc influx through calcium-permeable AMPA/kainate receptors also may be involved in intracellular zinc signaling (Takeda et al., 2007, 2009; Tamano and Takeda, 2011).

Calcium entry into cells via calcium-permeable AMPARs also may influence transmitter release. In the retina, calcium-permeable AMPARs, which are activated by glutamate released from rod bipolar cells, provide the influx of calcium needed to cause GABA release from A17 amacrine cells (Chávez et al., 2006). In the OB, calcium-permeable AMPARs have been shown to be the major receptor type underlying glutamatergic signaling in neural precursor cells (NPCs; Darcy and Isaacson, 2010). Under conditions of decreased transmitter uptake, glutamate spillover from synapses in the GCL can activate nonsynaptic NPC AMPARs and increase intracellular calcium levels (Darcy and Isaacson, 2010).

Recently, we showed that zinc potentiates AMPAR-mediated synaptic events (Blakemore et al., 2013). Using whole-cell current-clamp and voltage-clamp recording, we examined the effects of zinc on isolated AMPAR-mediated excitatory post-synaptic potentials (EPSPs) and post-synaptic currents (EPSCs) recorded from M/T cells and interneurons in primary cultures of rat OB neurons. Co-application of 100 μM zinc potentiated AMPAR-mediated EPSPs and/or EPSCs in 60% of M/T cells compared with only 23% of interneurons (Blakemore et al., 2013). Thus, consistent with our 2004 study, the frequency of zinc-mediated potentiation differed between cell types, which is likely due to variability in the AMPAR subunit composition. Also, consistent with our 2004 study, zinc perfusion reduced both EPSPs and EPSCs in subsets of M/T cells (10%) and interneurons (30%) and appeared to have no measureable effect in some M/T cells (30%) and interneurons (46%; Blakemore et al., 2013), further supporting the co-existence of cells with zinc-sensitive and zinc-insensitive AMPARs in the OB.

Together, these results have important implications for synaptic transmission, hence, odor processing. For example, zinc-mediated potentiation of the relatively small and brief AMPAR-mediated current could increase the typically minor role AMPARs play in reciprocal inhibition between M/T cells and interneurons (Isaacson and Strowbridge, 1998; Schoppa et al., 1998). In addition, we propose that calcium influx into cells through calcium-permeable AMPARs may be important at hyperpolarized potentials when the driving force for calcium entry is large but calcium flux via NMDARs is blocked by magnesium. Zinc-mediated potentiation of currents mediated by calcium-permeable AMPARs would increase calcium flux, potentially enhancing transmitter release.

Mitral and tufted cells within a glomerulus are synchronized by patterns of glutamate release from OSN (Carlson et al., 2000; Schoppa and Westbrook, 2001, 2002; Christie and Westbrook, 2006; Schoppa, 2006). This synchronization depends partly on AMPAR activation (Schoppa and Westbrook, 2002), generating oscillations with temporal and spatial features in response to odors. Glutamate release evokes the spatial patterns, but GABAergic neurons regulate the temporal patterns (Schoppa, 2006) that together underlie OB encoding of olfactory information. The results of our 2013 study extend our previous 2004 analyses of synaptic/extrasynaptic AMPARs (Blakemore and Trombley, 2004) by isolating the effects of zinc on synaptically activated AMPARs important to this OB circuit function. In M/T cells, AMPAR-mediated EPSPs were potentiated by zinc, thus, enhancing the frequency of synaptic summation (Blakemore et al., 2013). Such enhancement would facilitate neuronal synchronization and modify information processing by the OB.

### Zinc’s Effects on GABA_A_ Receptors

GABA_A_ receptors are ligand-gated ion channel receptors that flux chloride ions and mediate fast inhibitory transmission (Schofield et al., 1987; Barnard et al., 1988). Early electrophysiological evidence suggested the presence of functional GABA_A_ receptors in the OB (Trombley and Shepherd, 1993), and a variety of studies have shown GABA_A_ receptors to be expressed throughout the OB aside from the IPL (Laurie et al., 1992; Panzanelli et al., 2005; Ennis et al., 2007).

Westbrook and Mayer (1987) showed that zinc (5 μM) inhibited the response to GABA in cultured hippocampal neurons in a voltage-insensitive manner (Westbrook and Mayer, 1987). A variety of subsequent studies found that exogenously applied zinc mostly (but not always) inhibited GABA_A_ receptors (Smart et al., 1994).

Wang et al. (2001, 2002) demonstrated the presence of zinc-enriched GABAergic terminals in subpopulations of neurons in mouse spinal cord and cerebellar cortex. Ruiz et al. (2004) showed that both GABA and zinc are contained in the same hippocampal MF varicosities. Their finding that exogenously applied zinc depressed IPSCs recorded in hippocampal slices led them to conclude that endogenously released zinc modulates GABA_A_-receptor-mediated responses at zinc-containing MF synapses. Together, these results suggest that endogenous zinc may modulate inhibitory transmission.

We and others have demonstrated zinc-mediated modulation of GABA-mediated currents in OB neurons. Serafini et al. (1995) reported that GABA_A_-activated currents recorded in embryonic rat OB cells were partially inhibited by a wide range of zinc concentrations (10–1000 μM). We subsequently reported that zinc (100 μM) was an effective antagonist of GABA_A_-mediated currents in cultured rat OB neurons; the IC50 was 17 μM (Trombley and Shepherd, 1996). Consistent with zinc’s effect on exogenously evoked GABA_A_-mediated currents, we later showed that 100 μM zinc completely blocked GABA-mediated spontaneous inhibitory transmission (IPSPs) in OB neurons (Horning and Trombley, 2001).

Together, these results suggest that zinc modulates currents mediated by GABA_A_ receptors expressed by OB neurons. As one source of zinc-enriched terminals in the OB is OSN terminals contacting dendrites of M/T cells and PG cells (Jo et al., 2000; Blakemore et al., 2013), zinc released at these synapses may influence inhibition mediated by GABAergic JG cells.

Centrifugal pathway inputs from a variety of brain regions affect odor information processing in the OB. A second source of zinc-containing synaptic terminals in the mouse OB is centrifugal fibers projecting to granule cells and PG cells. Thus, zinc released from these centrifugal fibers may influence excitatory transmission at these synapses and subsequent inhibitory transmission mediated by GABAergic granule cells and PG cells.

### Zinc’s Effects on Glycine Receptors

The glycine receptor is a membrane-spanning protein that contains a Cl-selective ion channel, producing an inhibitory response when glycine binds that is similar to GABAergic inhibition. In the OB, immunoreactivity for glycine has been demonstrated in EPL and in granule cells (Pourcho et al., 1992), and robust labeling for glycine receptors exists (van den Pol and Gorcs, 1988; Weltzien et al., 2012). High levels of glycine are also found in the OB (Ennis et al., 2007), which inhibit rat OB cells (Trombley and Shepherd, 1994; Trombley et al., 1999).

Zinc is reportedly co-localized with glycine in inhibitory neurons in the lamprey and mouse spinal cord (Birinyi et al., 2001; Wang et al., 2001), and additional studies suggest that synaptic zinc is essential for the proper *in vivo* functioning of glycinergic neurotransmission in the spinal cord and brainstem (Hirzel et al., 2006). In rat spinal cord, low zinc concentrations (20 nM–1 μM) enhanced currents evoked by 100 μM glycine, while higher zinc concentrations (20–50 μM) caused a reversal of this potentiation, followed by progressive current inhibition (Bloomenthal et al., 1994).

Subsequent studies, including our own, have shown similar biphasic (potentiating and inhibiting), concentration-dependent effects of zinc on both native and recombinant glycine receptors (Laube et al., 1995; Trombley and Shepherd, 1996; Lynch et al., 1998; Harvey et al., 1999; Lynch, 2004; Trombley et al., 2011). Zinc’s potentiating and inhibiting concentrations are often reported as <10 μM and >10 μM, respectively (Lynch, 2004). However, the glycine receptor’s subunit composition introduces variability in zinc’s concentration-dependent effects (Miller et al., 2005a), and we have observed biphasic effects in the OB at higher concentrations of zinc (Trombley and Shepherd, 1996; Trombley et al., 2011).

Results from our 1996 study advance the results of previous studies by suggesting that the effects of zinc on glycine receptors in the rat OB only occur with use of low concentrations of glycine, which do not desensitize the receptors. We showed that 100 μM zinc potentiated the current when a non-desensitizing concentration of glycine (30 μM) was used. A higher concentration of zinc (1 mM) inhibited currents evoked by 30 μM glycine. However, zinc (up to 1 mM) had no effect on the steady-state, desensitized component of the current evoked by a high concentration of glycine (300 μM; Trombley and Shepherd, 1996).

Because the synaptic cleft concentration of glycine is greater than 2 mM (Beato, 2008), glycine receptors are rapidly saturated and transition to a desensitized state during synaptic transmission. With low mM concentrations, glycine receptors progressively desensitize with even short (1 ms) pulses of glycine when applied in excess of 1 Hz (Rigo and Legendre, 2006). Of additional significance, it has been recently demonstrated that pulse applications, within a normal physiological frequency, cause significant receptor desensitization and that much of this happens in between the pulses when glycine is absent (Papke et al., 2011). Consequently, glycine receptors undergo significant desensitization during normal physiological conditions.

Zinc’s inhibition of glycine receptors is reportedly due to stabilization of the closed state of the receptor (Lynch, 2004). Therefore, we hypothesized that high micromolar glycine concentrations may result in the occupation of all glycine binding sites and a conformational change in the receptor (to a desensitized state) that prevents zinc binding or the effects of bound zinc. To test this hypothesis, we continuously applied 300 μM zinc during intermittent pulse application of 300 μM glycine; we found that such pre-application of zinc slowly inhibited currents evoked by desensitizing concentrations of glycine recorded in cultured rat OB neurons (Trombley et al., 2011). Another protocol revealed increasing zinc-mediated inhibition of this current with increasing durations of zinc exposure (Trombley et al., 2011).

Several types of receptors and ion channels allow synaptically released zinc to enter the postsynaptic neuron (Koh and Choi, 1994; Sensi et al., 1997, 1999; Kerchner et al., 2000; Weiss and Sensi, 2000; Tamano and Takeda, 2011), where it could interact with an intracellular domain of the glycine receptor. To explore this hypothesis, we compared the fluorescence of neurons loaded with a zinc-sensitive probe (Newport Green) under control conditions (no added zinc) to conditions in which 300 μM extracellular zinc was applied for 60 s. Compared with control neurons, neurons exposed to 300 μM zinc were more robustly labeled, confirming that extracellular application of zinc results in elevated intracellular zinc (Trombley et al., 2011).

To further explore our hypothesis that intracellular zinc contributes to inhibition, we included several types of zinc chelators (10 mM EDTA, 100 μM dipicolinic acid, or 100 μM 1,10-phenanthroline) in our recording electrode and evoked a glycine current with application of 300 μM glycine; 300 μM zinc was then preapplied for 60 s and another glycine-evoked current was measured. None of the chelators prevented the inhibitory effects of externally applied zinc on the current. These results may suggest that the chelation process was too slow to prevent an intracellular effect of zinc or that the increase in intracellular zinc did not affect inhibition. However, the most likely explanation for this finding is that an extracellular binding site mediates inhibition by externally applied zinc (Trombley et al., 2011). Consistent with this notion, the site(s) for zinc’s inhibitory actions on the glycine receptor appear(s) appear to be located on the external face of the alpha subunit (Laube et al., 1995, 2000; Nevin et al., 2003; Miller et al., 2005a).

To further examine zinc’s possible intracellular effects, a range of zinc concentrations (1, 10, 100 μM) was added to the recording electrode (Trombley et al., 2011). All concentrations of zinc rapidly increased the amplitude of the current evoked by 300 μM glycine, which was followed by a slight decrease in the current. Inclusion of a chelator with zinc in the electrode blocked these effects of intracellular zinc. These results represent the first report that glycine receptor-mediated currents can be potentiated by low concentrations of intracellular zinc.

Previous studies have suggested that the zinc potentiation site(s) on the glycine receptor is/are located on the external face of the alpha subunit’s N-terminal domain (Laube et al., 1995, 2000; Lynch, 2004; Miller et al., 2005b). However, our finding that low concentrations of intracellular zinc potentiate glycine receptor-mediated currents also suggest the presence on an internal zinc binding site that mediates potentiation by zinc. Several studies have shown that the glycine receptor contains several amino acids (His, Glu, Asp) on the intracellular loops capable of binding zinc (Laube et al., 2002; Lynch, 2004; Miller et al., 2005b).

Together, these results illustrate the complexity of zinc’s effects on glycine receptors as well as the many interacting factors that influence glycinergic inhibition; these factors include the receptor subunit composition, the state of the receptor, the timing of exposure to zinc and glycine, the concentration of zinc exposure, and whether zinc gains intracellular access. Each of these factors would significantly alter the efficacy of glycinergic transmission in the OB.

## Zinc Modulation of Voltage-Gated Ion Channels with A Focus on the OB

Zinc modulation of voltage-gated sodium, potassium and calcium channels has been shown by us and others. The below discussion focuses on several types of voltage-gated ion channels found in the OB.

### Zinc’s Effects on Voltage-Gated Calcium Channels

Voltage-gated calcium channels are ion channels found in the membranes of excitable cells that are primarily permeable to calcium. Several different types of high-voltage-activated (HVA) calcium channels (L-type, N-type, P/Q-type) exist, which are variously distributed in the central and peripheral nervous systems. The T-type calcium channel is a low-voltage-activated (LVA) channel, which is also expressed by neurons in some brain regions (Mathie et al., 2006).

At physiologic or resting membrane potentials, voltage-gated calcium channels are usually closed. However, at depolarized membrane potentials they open, permitting calcium to enter the cell and triggering the release of neurotransmitter into the synaptic cleft. Voltage-gated and ligand-gated (NMDA and AMPA) calcium channels are also involved in intracellular calcium signaling (Weiss and Sensi, 2000; Foster, 2007), which is influenced by zinc (Tamano and Takeda, 2011).

Early studies demonstrated inhibitory effects of zinc on voltage-gated calcium currents. Zinc was shown to block voltage-gated calcium-channel currents in cultured rat dorsal root ganglion cells (Büsselberg et al., 1994). A later study examined the effects of zinc on voltage-gated calcium channels in acutely dissociated neurons from the horizontal limb of the diagonal band of Broca (hDBB; Easaw et al., 1999), a region enriched in zinc-positive fibers (Mandava et al., 1993). Zinc (50 μM) reversibly attenuated calcium-channel currents recorded in rat hDBB neurons by approximately 85%, with varying effects on L-, N- and P-type HVA calcium-channel currents (Easaw et al., 1999). Zinc (6–200 μM) was later shown to block total HVA calcium-channel currents in pyramidal neurons acutely dissociated from rat piriform cortex, with inhibition of all pharmacological components (L-, N-, P/Q- and R-type) of these currents (Magistretti et al., 2003).

In regard to zinc modulation, Mathie et al. (2006) claimed that the T-type or LVA calcium channels are arguably the most important group. Three channel isoforms (Cav3.1, Cav3.2 and Cav3.3) comprise this family, and neurons in some regions of the OB express these channels (Talley et al., 1999; Liu and Shipley, 2008b; Johnston and Delaney, 2010). Takahashi and Akaike (1991) showed zinc-mediated inhibition (IC50 of 20 μM) of the T-type calcium channel in CA1 pyramidal cells from rat hippocampus. A later study showed zinc-mediated inhibition of T-type calcium channels in rat dorsal root ganglion cells, with 20 μM zinc generating a block exceeding 80% (Büsselberg et al., 1992). The IC50 for zinc inhibition of N- and L-type channels was higher (69 μM). Another study found that zinc blocked 3 types of recombinant T-type calcium channels in a concentration-dependent manner, with a higher affinity for Cav3.2 channels (IC50 2.3 μM; Jeong et al., 2003).

Evidence from a variety of studies suggests that zinc modulation of calcium channels affects calcium influx and transmitter release. A study in rat dorsal root ganglion neurons found that low concentrations (10–100 nM) of zinc evoked an extracellular calcium influx through L-, N- and T-type voltage-gated calcium channels, thus, increasing the release of substance P; higher concentrations (1–100 μM) of zinc attenuated or completely masked these responses (Tang et al., 2009). In the hippocampus and amygdala, high-frequency stimulation with the membrane-impermeable zinc chelator CaEDTA present enhanced exocytosis (Minami et al., 2006; Takeda et al., 2007, 2010). Also in the hippocampus, application of the zinc chelator TPEN during LTP enhanced presynaptic calcium signals without affecting synaptic transmission (Quinta-Ferreira and Matias, 2004). Thus, it has been proposed that endogenous released zinc may control calcium influx through voltage-gated calcium channels (Tamano and Takeda, 2011). Furthermore, voltage-gated calcium channels and calcium-permeable AMPA/kainate receptors allow released zinc to be taken up into the presynaptic and postsynaptic neurons, where it may further modulate calcium influx and serve intracellular zinc signaling functions (Tamano and Takeda, 2011).

In our 2001 study (Horning and Trombley, 2001), we examined the effects of zinc on voltage-gated calcium-channel currents recorded from cultured rat OB neurons. We hypothesized that the effects of zinc on calcium-channel currents could contribute to zinc’s inhibitory effects on excitatory transmission (Trombley et al., 1998; Horning and Trombley, 2001). We induced calcium-channel currents with kinetic profiles typical of HVA channels in M/T cells. Zinc (100 μM) significantly inhibited these calcium-channel currents by about 60%.

Various investigators have reported the presence and actions of T-type channels in the OB. Pignatelli et al. (2005) demonstrated that an LVA (T-type) calcium channel current plays a role in the autorhythmicity of mouse OB dopaminergic neurons in the GL. The effects of zinc released from OSN terminals and centrifugal fibers in the GL on this current could alter the role of these dopaminergic neurons in glomerular circuits.

Mitral cells express LVA Cav3.3 (T-type) channels on their distal apical tuft dendrites (Johnston and Delaney, 2010). In the mouse OB, calcium influx from these channels influences both asynchronous glutamate release from these dendrites and feedback inhibition from PG cells (Fekete et al., 2014). Thus, zinc released from OSN terminals and centrifugal fibers could inhibit these calcium channels and alter reciprocal inhibition at mitral cell-PG cell synapses.

ET cells have a prominent calcium conductance that is activated at approximately −50 mV and has biophysical and pharmacological features of both T- and L-type calcium currents (Liu and Shipley, 2008b). These investigators also demonstrated that L- and/or T-type-mediated LVA calcium currents amplified both subthreshold and suprathreshold synaptic responses in ET cells in response to ON input in mouse OB (Liu and Shipley, 2008a). Again, zinc released from OSN terminals and centrifugal fibers could modify these responses.

Our 2001 results, suggesting that micromolar concentrations of zinc inhibit HVA calcium channels, also may have important implications to bulb function. In the OB, voltage-gated calcium (P/Q- and N-type) channels are important to ON activation of OB neurons (Isaacson and Strowbridge, 1998). Therefore, zinc released by OSNs in the GL may influence this activation. Calcium influx via NMDARs and voltage-gated calcium (P/Q- and N-type) channels close to granule cell-mitral cell dendrodendritic synapses is associated with transmitter (GABA) release (Isaacson and Strowbridge, 1998; Chen et al., 2000; Halabisky et al., 2000; Isaacson, 2001; Egger et al., 2003, 2005). Thus, zinc released from centrifugal fiber terminals contacting granule cells may inhibit voltage-gated calcium channels expressed by these neurons, affecting excitability and/or indirectly influencing transmitter release.

### Zinc’s Effects on Voltage-Gated Sodium Channels

Voltage-gated sodium channels are composed of a pore-forming alpha subunit and two auxiliary beta subunits that modulate channel gating (Catterall, 2000; Catterall et al., 2005). The individual voltage-gated sodium channel subtypes are defined by the nine different alpha channel subunits (Na_v_1.1–Na_v_1.9; Deuis et al., 2017). Several types of these subunits (e.g., Na_v_1.1, Na_v_1.6, Na_v_ 1.7) are heterogeneously expressed in the rat olfactory system (Lorincz and Nusser, 2008; Ahn et al., 2011).

Whereas most voltage-gated sodium channels are highly sensitive to blockade by tetrodotoxin (TTX), some (e.g., Na_v_1.5, Na_v_1.8 and Na_v_1.9) are much less sensitive to TTX (Goldin, 2001; Mathie et al., 2006). A comparison of the sensitivity of sodium channels to zinc revealed potent inhibition of TTX-resistant channels by zinc (IC50 of 50 μM) compared to TTX-sensitive channels (IC50 of 2 mM; Frelin et al., 1986). Whereas Na_v_1.5 channels are frequently characterized as “‘cardiac”’ sodium channels, some neurons also express these channels (Mathie et al., 2006).

In rat medial entorhinal cortical neurons, both TTX-sensitive and TTX-resistant sodium channels were found, with the latter being highly sensitive to zinc (IC50 of around 9 μM; White et al., 1993; Mathie et al., 2006). At a higher concentration (around 300 μM), zinc inhibited inward TTX-resistant sodium channels in dorsal root ganglion neurons, which chiefly express Na_v_1.8 and Na_v_1.9 channels (Kuo et al., 2004).

In our 2001 study, we also examined the effects of zinc on voltage-gated sodium channels in cultured rat OB neurons (Horning and Trombley, 2001). We hypothesized that our observed suppression of excitatory transmission by zinc (Trombley et al., 1998; Horning and Trombley, 2001) may be mediated, in part, by actions on voltage-gated sodium channels, which are largely responsible for the upstroke of an action potential. Sodium currents were evoked under voltage-clamp conditions, and 100 μM zinc significantly decreased the sodium current by approximately 20%.

In our experiments in the OB, application of 1 μM TTX blocked these voltage-gated sodium currents; this finding confirms that these currents, which were inhibited by 100 μM zinc, were mediated by typical TTX-sensitive sodium channels. It is plausible that the OB contains a mixture of TTX-sensitive and TTX-resistant sodium channels. Given the findings of previous investigators, it is possible that lower concentrations of synaptic zinc may modulate TTX-resistant sodium channels in the OB.

Delgado et al. (2006) subsequently investigated the effects of several metals including zinc on TTX-sensitive voltage-dependent inward sodium currents in toad OSNs (Aedo et al., 2007). At a very low concentration (0.1 μM), zinc increased the amplitude and accelerated the kinetics of activation and inactivation. At a much higher concentration (100 μM), zinc inhibited the inward sodium current by 33% (Delgado et al., 2006). These investigators also found that low concentrations (1–50 μM) of zinc increased the neuronal firing rate in a concentration-dependent manner, while higher concentrations (100–500 μM) of zinc decreased the firing rate (Aedo et al., 2007). These findings are consistent with our findings in the OB.

The observed effects of zinc on voltage-gated sodium channels have implications to OB function. When rat OB mitral cells are held near threshold, they can generate rebound spikes that require recovery of subthreshold TTX-sensitive sodium channels (Balu and Strowbridge, 2007). These investigators further showed that currents mediated by these channels contribute to rebound spikes following granule cell-mediated IPSPs by enhancing subthreshold excitatory events. Thus, zinc-mediated inhibition of these voltage-gated sodium channels could influence the bidirectional control of spike output from the OB.

ET cells have a TTX-sensitive persistent sodium current (Hayar et al., 2004b; Liu and Shipley, 2008b), which is necessary for spontaneous burst initiation in ET cells (Hayar et al., 2004b). Liu and Shipley (2008a) investigated the actions of this current on excitatory synaptic transmission from ON to ET cells in mouse OB. Collectively, their results suggest that this persistent sodium current amplifies subthreshold postsynaptic excitatory responses (EPSPs) and produces temporal summation. Dopamine neurons in the mouse OB also have a mostly TTX-sensitive persistent sodium current that contributes to autorhythmicity of these neurons (Pignatelli et al., 2005). Given the well-documented importance of both ET and DA cells in regulating glomerular activity, zinc-mediated inhibition of this current could alter several critical glomerular circuit functions underlying odor information processing.

### Zinc’s Effects on Voltage-Gated Potassium Channels

Voltage-gated potassium channels are the largest family of voltage-gated ion channels, consisting of ~40 subunits that form multiple distinct channels with different functional properties (Gutman et al., 2003, 2005; Nusser, 2009). They influence neuronal excitability in numerous ways including being responsible for the repolarization phase of action potentials and regulating action potential frequency. The focus of this discussion is on two subtypes: delayed rectifier and A-type potassium channels.

A variety of delayed-rectifier and A-type potassium channel subunits (e.g., Kv1.1, Kv1.2, Kv1.3, Kv1.4, Kv3.1, Kv3.2, Kv4.2, and Kv4.3) are heterogeneously expressed in the OB (Veh et al., 1995; Fadool et al., 2004; Kollo et al., 2008; Lorincz and Nusser, 2008; Boda et al., 2012). Several voltage-gated potassium channel subunits (Kv2.1, Kv3.1b, Kv4.3) are found in a high percentage (70%–95%) of deep SA cells (Eyre et al., 2009). It has been shown that intracellular zinc and calcium released in response to oxidative stimuli play a role in the insertion of the Kv2.1 channel into the plasma membranes of cortical neurons, detected by significant enhancement of the delayed rectifier potassium currents after brief exposure to apoptogenic stimuli (McLaughlin et al., 2001; Pal et al., 2003, 2006; Redman et al., 2007, 2009; McCord and Aizenman, 2013). This current enhancement leads to a loss of intracellular potassium that regulates processes involved in neuronal apoptosis (McCord and Aizenman, 2013).

Various potassium channels, including A-type and delayed-rectifier channels, are present or are probably expressed on granule cell dendrites in the OB (Veh et al., 1995; Schoppa and Westbrook, 1999; Bywalez et al., 2015), where A-type potassium channels play an important role in granule cell signaling (Schoppa and Westbrook, 1999). Bywalez et al. (2015) recently concluded that delayed-rectifier currents likely play a role in local repolarization in granule cell dendritic spines. Other types of potassium channels have important functions in the OB; these include large-conductance calcium–dependent K currents (I_BK_) in ET cells that terminate spontaneous bursting (Liu and Shipley, 2008b) and small-conductance calcium-activated potassium channels (SK) activated either by calcium channels or NMDARs in mitral cell dendrites that influence action potential firing and dendrodendritic inhibition (Maher and Westbrook, 2005). Thus, effects of zinc on potassium channels in the OB would have important implications to OB function.

#### Zinc’s Effects on Transient and Steady-State Outward Potassium Currents

Zinc inhibition of A-type channels was first shown in cultured rat sympathetic neurons (Constanti and Smart, 1987). It was subsequently shown that zinc (1 μM–1 mM) markedly alters the voltage dependence of activation and inactivation for A-type channels in rat cerebellar granule cells (Bardoni and Belluzzi, 1994). Zinc-induced shifts in the steady-state inactivation and activation curves to more positive potentials increased the peak current amplitude near resting membrane potentials and decreased the peak current at hyperpolarized potentials (Bardoni and Belluzzi, 1994). In another study, zinc (2 μM–1000 μM) modulated the gating (slowed activation) of delayed-rectifier channels cloned from rat and human tissues in a concentration-dependent manner (Harrison et al., 1993).

In our 2001 study, we also examined the effects of zinc on voltage-gated potassium channels in cultured rat OB neurons (Horning and Trombley, 2001). First, outward currents consistent with the kinetics of a delayed rectifier-type current were evoked by clamping neurons at −50 mV and stepping to +20 mV for 100 ms. Zinc (100 μM) significantly inhibited the steady-state current amplitudes but potentiated the peak current amplitudes in most neurons examined. This latter effect was due to the activation of a transient A-type outward current that was not observed in the absence of zinc.

The transient A-type potassium current helps mediate the interspike interval and is mostly activated by a depolarizing event that follows hyperpolarization. We examined this current by holding neurons at a hyperpolarizing potential (−90 mV) and stepping them to +20 mV for 100 ms, which activated both the A-type and delayed rectifier potassium currents. Zinc (100 μM) significantly inhibited both the transient component (A-type) and the steady-state component (delayed rectifier-type) of the potassium currents evoked under these conditions.

Our results suggest that zinc’s effects on potassium channels are influenced not only by the type of potassium channel but also by the membrane voltage. While zinc inhibited the delayed rectifier-type outward current at all voltages examined, its effects on the transient A-type current were more complex. Zinc (100 μM) inhibited A-type currents when the membrane voltage was hyperpolarized but enhanced the current when the membrane was at or depolarized to a typical resting potential where most A-type channels are inactivated.

Consistent with our findings in the OB, 50 μM zinc reduced the amplitude of delayed-rectifier currents and increased the peak amplitude of A-type currents in rat hDBB neurons (Easaw et al., 1999). Our observed voltage-dependent effects of zinc on A-type channels in OB neurons are also similar to the above-described findings in cerebellar neurons (Bardoni and Belluzzi, 1994).

Another study examined the effects of zinc on A-type currents in PG cells in the rat OB. Voltage-clamp analyses of PG cells revealed two subpopulations based on the characteristics of their potassium conductances (Puopolo and Belluzzi, 1998). The first group of PG cells displayed delayed-rectifier and fast transient A-type currents of similar amplitude. The second group of PG cells had a large A-type current and a small or absent delayed-rectifier current. In this study, zinc (10–300 μM) affected A-type, but not delayed-rectifier, channels. When the inactivation of A-type channels was removed with a hyperpolarizing step to −120 mV, zinc caused a slight reduction in the current amplitude Similar to our results, the effect of zinc on the A-type current was potentiation when the membrane was depolarized starting from physiological holding potentials (e.g., −50 mV; Puopolo and Belluzzi, 1998).

#### Zinc’s Effects on Potassium Channels and Repetitive Firing

As the delayed rectifier-type current is involved in the repolarization phase of action potentials, its inhibition would likely reduce the rate of repetitive firing. Potentiation of the A-type outward current would slow the depolarization rate, thus, also reduce the repetitive firing rate. Consistent with this prediction, at a holding potential of −50 mV, 100 μM zinc reduced neuronal firing in all neurons examined by about 50% (Horning and Trombley, 2001). At a holding potential of −90 mV, 100 μM zinc decreased the number of action potentials fired by most (~70%) neurons by about 63%. However, zinc also increased action potential frequency in a significant proportion (~30%) of neurons at this holding potential. Thus, zinc appears to modulate repetitive firing in a voltage-dependent manner, with some variability perhaps due to the diversity of potassium channel expression among OB neurons.

The effects of zinc on potassium currents in the OB have implications to bulb function. There exists a delay in the onset of action potential firing in M/T cells, which may be attributable to A-type potassium currents (Chen and Shepherd, 1997). As zinc-containing OSNs synapse with mitral, tufted, and PG cells, zinc co-released with glutamate may affect glomerular circuit activity via effects on these A-type currents. In addition, zinc’s ability to dramatically alter the excitability of the subset of PG cells that express primarily the A-type channel (Puopolo and Belluzzi, 1998; Fogli Iseppe et al., 2016) could significantly alter glomerular circuit function via regulation of feedback and feedforward inhibition from these cells.

A-type currents are also responsible for a delay in action potential firing in granule cells, thus, influence the timing of reciprocal inhibition (Schoppa and Westbrook, 1999). As granule cells appear to be targets of zinc-containing centrifugal fibers, zinc’s opposing actions on A-type currents also may indirectly affect reciprocal inhibition mediated by these cells.

## Potential Effects of Zinc Signaling on Behavior

### Results from ZnT3 KO Mice

Some previous studies have examined the effects of application of zinc or zinc chelators on behavior. Intrathecal injection of zinc or zinc chelators (CaEDTA, dipicolinic acid) altered nociceptive and antinociceptive activity in mice (Larson and Kitto, 1997, 1999). Infusion of the zinc chelator diethyldithiocarbamate into the hippocampus disrupted spatial-working memory (Frederickson et al., 1990) and spatial learning (Lassalle et al., 2000) in rodents. In a later study, diethyldithiocarbamate or CaEDTA infusion into the hippocampus of mice disrupted the acquisition and consolidation of contextual fear conditioning, without affecting retrieval (Daumas et al., 2004). However, the development of the ZnT3 KO mouse has provided a novel tool to test the hypothesis that zinc signaling may modulate behavior, as reviewed by McAllister and Dyck (2017).

Early behavioral characterization of ZnT3 KO mice by Cole et al. (2001) revealed minimal abnormalities in a variety of behaviors including those involving motor coordination, thermal nociception, olfaction, auditory startle response, anxiety, and certain forms of learning and memory (e.g., passive avoidance, fear conditioning, spatial learning, working and reference memory; Cole et al., 2001; McAllister and Dyck, 2017). More recent evidence showed differences between ZnT3 KO mice and control mice in certain behavioral tests assessing fear conditioning and memory (Martel et al., 2010) and social memory (Martel et al., 2011). Differences between ZnT3 KO mice and control mice in performance on the standard water maze further suggest altered spatial navigation and memory in ZnT3 KO mice (Martel et al., 2011; McAllister and Dyck, 2017). A Sindreu et al. (2011) study found deficits in contextual discrimination and spatial working memory in ZnT3 KO mice. Perhaps the greatest evidence of behavioral abnormalities in ZnT3 KO mice comes from a recent study by Yoo et al. (2016) who observed sex-dependent behavioral abnormalities in young male ZnT3 KO mice consistent with autistic behavior, which included reduced social interaction, decreased novelty seeking behavior, increased repetitive behavior and reduced locomotion.

Little is known about the potential effects of zinc modulation in the OB on olfactory behavior. In the study by Cole et al. (2001), olfaction in ZnT3 KO mice was measured in two ways: (1) measurement of latency to find food hidden beneath bedding to assess gross olfactory deficits; and (2) a conditioning paradigm requiring mice to associate a non-noxious odor (peppermint) with an aversive compound (quinine) to assess the olfactory threshold (Cole et al., 2001). ZnT3 KO and control mice had similar food-recovery latencies and olfactory thresholds. However, they acknowledge there are some limitations to this study. For example, the tests employed may not be the most sensitive to neuromodulation by zinc. As they indicated, KO mice also may compensate during development by altering neuronal anatomy, neurotransmitter release, or receptor sensitivity (Cole et al., 2001). Thus, they suggest that zinc may be an effective neuromodulator in wild-type mice, but KO mice could compensate easily for its absence. Furthermore, some behavioral abnormalities are strongly sex-dependent (Yoo et al., 2016), and this variable was not examined in the olfactory studies by Cole et al. (2001).

### Results from Progranulin-Deficient (KO) Mice

Progranulin is a protein with both neuroprotective and immunological properties (Rademakers et al., 2012), and its functions have been proposed to include protein homeostasis (Hardt et al., 2017). As progranulin deficiency in humans is linked to frontotemporal lobar degeneration (Baker et al., 2006; Cruts et al., 2006). Hardt et al. (2017) assessed psychopathology and chronic pain in progranulin-deficient (KO) mice. They present novel data suggesting that defective neuronal zinc transport may play a role in psychopathology associated with progranulin deficiency, which includes abnormalities in olfactory behavior.

In addition to assessing behaviors related to memory, emotions (e.g., anxiety, depression) and nociception, Hardt et al. (2017) assessed odor preference and discrimination in progranulin KO and control mice. Interest and preferences of odors prior to and following peripheral nerve injury were assessed using cinnamon (familiar odor) and vanilla (novel odor). Whereas control animals showed no loss of interest in pleasant odors after nerve injury, progranulin KO mice increased visit frequency of odors after nerve injury, leading to longer traveling paths. Odor discrimination was assessed using rose (novel odor) vs. vanilla (familiar odor). In contrast to controls, progranulin KO mice did not discriminate between these odors.

Due to the abnormal odor-related behavior in progranulin KO mice, these investigators performed deep proteome analyses of the prefrontal cortexes and OBs of progranulin KO and control mice (Hardt et al., 2017). This revealed progranulin-dependent changes in proteins involved in synaptic transport, with a loss of nuclear and synaptic zinc transporters (ZnT9/Slc30a9; ZnT3/Slc30a3) associated with progranulin deficiency. Western Blot analysis of the OB, prefrontal cortex and hippocampus revealed a complete loss of ZnT3/Slc30a3 in progranulin KO mice along with decreased plasma zinc levels (Hardt et al., 2017). Collectively, these results suggest that previously unrecognized zinc-dependent molecular mechanisms underlie progranulin-related loss of function, including deficits in odor preference and discrimination.

## Concluding Remarks

While most attention has focused on zinc’s effects on neuronal excitability and synaptic transmission in other regions of the CNS, comparatively little is known about zinc’s effects in the OB (see Supplementary Tables S1, S2). This opens the door for many future directions of research into mechanisms by which zinc could modulate odor information processing.

For example, NMDARs have a multitude of important functions, ranging from regulating mitral/tufted cell spiking to dendrodendritic inhibition. GABAergic transmission is also critical to both intra- and inter-glomerular inhibition and synchronization. It is clear that zinc is an effective modulator of both of these receptor types, but both the circuit details and the broad implications of such modulation remain to be elucidated.

The impact of zinc on OB circuit behavior due to modulation of AMPARs and glycine receptors is much more complex. This is due to the exceptional diversity of zinc’s modulatory effects (potentiation and/or inhibition) on subpopulations of these receptors in the OB, which are influenced by factors including the receptor molecular composition, the extracellular environment, the (desensitized) state of the receptor, as well as intracellular and extracellular sites of zinc action.

Zinc’s effects on voltage-gated ion (e.g., calcium, sodium, potassium) channels add another significant dimension, as numerous subtypes of these channels have important regulatory function on OB neuron excitability and network function.

Substantial progress has been made in the initial characterization of what zinc can do to modify excitability and synaptic transmission. However, it simply just scratches the surface of what is needed to be known to fully appreciate the impact of zinc on odor processing by the OB. This also highlights a significant aspect of using the OB to gain a broader understanding of the role of zinc as a neuromodulator. While the bulk of what is known about zinc-mediated neuromodulation comes from other brain regions (e.g., hippocampus), the functions of those brain regions and the subcircuits contained within (e.g., MFs) are not entirely well defined. A major advantage of using the OB to investigate the actions of zinc is that we know precisely the function of the OSN → OB glomerular network (where most of the zinc is located), that is—the processing of odor information. We would be hard pressed to identify a brain region more lucrative for identifying mechanisms involved in modifying/regulating information processing. This, in itself, makes the OB a unique tool for understanding the significance of zinc-mediated neuromodulation in the CNS.

## Author Contributions

Both authors contributed equally to this manuscript. LJB wrote the first draft of the manuscript and PQT edited this draft and contributed to all subsequent drafts. All authors agreed upon the final version of this manuscript.

## Conflict of Interest Statement

The authors declare that the research was conducted in the absence of any commercial or financial relationships that could be construed as a potential conflict of interest.

## Abbreviations

AMPA: alpha-amino-3-hydroxy-5-methyl-4-isoxazolepropionic acid
AMPARs: AMPA receptors
CNS: central nervous system
EPL: external plexiform layer (olfactory bulb)
EPSC: excitatory postsynaptic current
EPSP: excitatory postsynaptic potential
ET cells: external tufted cells
GABA: gamma-aminobutyric acid
GCL: granule cell layer (olfactory bulb)
GL: glomerular layer (olfactory bulb)
hDBB: horizontal limb of the diagonal band of Broca
HVA: high-voltage activated
IC50: half maximal inhibitory concentration
IPL: internal plexiform layer (olfactory bulb)
IPSP: inhibitory postsynaptic potential
JG cells: juxtaglomerular cells
KO: knockout
LTP: long-term potentiation
LVA: low-voltage activated
MCL: mitral cell layer (olfactory bulb)
MF: mossy fiber (hippocampus)
M/T cells: mitral/tufted cells
NMDA: N-methyl-D aspartate
NMDARs: NMDA receptors
OB: olfactory bulb
ONL: olfactory nerve layer (olfactory bulb)
OSN: olfactory sensory neuron
PG cells: periglomerular cells
SA cells: short axon cells
TPA: tris(2-pyridylmethyl)amine
TPEN: *N,N,N′,N′*-tetrakis (2-pyridylmethyl)ethylenediamine
TTX: tetrodotoxin.
