# Supplementary material for: Zinc as a Neuromodulator in the Central Nervous System with a Focus on the Olfactory Bulb
Source: Front Cell Neurosci. 2017 Sep 21;11:297. doi: 10.3389/fncel.2017.00297 (PMC5627021; doi:10.3389/fncel.2017.00297)
Supplement: Supplementary file 1 [file Table_1.docx]

Laura J. Blakemore and Paul Q. Trombley: “Zinc as a Neuromodulator in the Central Nervous System with a Focus on the Olfactory Bulb”

Table 1. Zinc effects on amino acid receptors expressed by olfactory bulb neurons.

| **Receptor type** | **Effects of zinc** |
| --- | --- |
| NMDA | 1 µM zinc had little effect on currents evoked by 100 µM NMDA (Trombley and Shepherd, 1996).  30 µM and 100 µM zinc inhibited currents evoked by 100 µM NMDA in cultured rat OB neurons (Trombley and Shepherd, 1996; Trombley et al., 1998). The IC50 was 19 µM (Trombley and Shepherd, 1996).  100 µM zinc reduced glutamate-mediated spontaneous excitatory activity (EPSPs) in cultured rat OB neurons (Horning and Trombley, 2001). |
| AMPA | 30 µM and/or 100 µM zinc potentiated, whereas 1 mM zinc inhibited, currents evoked by 50 µM AMPA in a subset of cultured rat OB neurons (Blakemore and Trombley, 2004).  Effects of a range of concentrations of zinc (30 µM, 100 µM, and 1 mM) on AMPAR receptor-mediated currents recorded from individual OB neurons were biphasic, uniphasic, or absent (Blakemore and Trombley, 2004).  Modulation of AMPAR-mediated currents was observed in a greater percentage of M/T cells than interneurons at low (30 µM, 100 µM) but not high (1 mM) zinc concentrations. However, zinc’s effect on AMPAR-mediated currents was greater in magnitude in interneurons than in M/T cells (Blakemore and Trombley, 2004).  100 μM zinc potentiated AMPA receptor-mediated EPSPs and/or EPSCs in greater proportion of M/T cells (60%) than interneurons (23%) in cultured rat OB neurons (Blakemore et al., 2013). |
| GABA_A_ | 1 µM zinc had little effect on currents evoked by 30 µM GABA (Trombley and Shepherd, 1996).  30 µM and 100 µM zinc inhibited currents evoked by 30 µM GABA in cultured rat OB neurons (Trombley and Shepherd, 1996; Trombley et al., 1998). The IC50 was 17 µM (Trombley and Shepherd, 1996).  A wide range of zinc concentrations (10-1000 µM) inhibited GABA_A_-mediated currents in acutely dissociated rat OB cells (Serafini et al., 1995).  100 µm zinc reduced spontaneous inhibitory activity (IPSPs) mediated by GABA_A_ receptors on cultured rat OB neurons (Horning and Trombley, 2001). |
| Glycine | 30 μM and 100 µM zinc potentiated, while 300 µM and 1 mM zinc inhibited, currents evoked by a low non-desensitizing concentration (30 µM) of glycine in cultured rat OB neurons (Trombley and Shepherd, 1996; Trombley et al., 2011).  Co-application of zinc (30 μM, 300 μM, 1 mM) had no effect on currents evoked by a high desensitizing concentration (300 µM) of glycine (Trombley and Shepherd, 1996; Trombley et al., 2011). However, pre-application of zinc (300 μM) resulted in a slowly developing inhibition (50%) of currents evoked by a desensitizing concentration of glycine, suggesting the receptor has to be in a desensitized conformation prior to zinc binding to prevent actions of zinc (Trombley et al., 2011).  Low concentrations of intracellular zinc (1 μM, 10 μM, and 100 μM zinc included in the recording electrode) potentiated currents evoked by application of 300 μM glycine (Trombley et al., 2011). |

EPSCs, excitatory postsynaptic currents; EPSPs, excitatory postsynaptic potentials; IPSPs, inhibitory postsynaptic potentials; M/T, mitral/tufted; OB, olfactory bulb

**References**

Blakemore, L.J., Tomat, E., Lippard, S.J., and Trombley, P.Q. (2013). Zinc released from olfactory bulb glomeruli by patterned electrical stimulation of the olfactory nerve. *Metallomics* 5(3)**,** 208-213. doi: 10.1039/c3mt20158a.

Blakemore, L.J., and Trombley, P.Q. (2004). Diverse modulation of olfactory bulb AMPA receptors by zinc. *Neuroreport* 15(5)**,** 919-923.

Horning, M.S., and Trombley, P.Q. (2001). Zinc and copper influence excitability of rat olfactory bulb neurons by multiple mechanisms. *J Neurophysiol* 86(4)**,** 1652-1660.

Serafini, R., Valeyev, A.Y., Barker, J.L., and Poulter, M.O. (1995). Depolarizing GABA-activated Cl- channels in embryonic rat spinal and olfactory bulb cells. *J Physiol* 488 ( Pt 2)**,** 371-386.

Trombley, P.Q., Blakemore, L.J., and Hill, B.J. (2011). Zinc modulation of glycine receptors. *Neuroscience* 186**,** 32-38. doi: 10.1016/j.neuroscience.2011.04.021.

Trombley, P.Q., Horning, M.S., and Blakemore, L.J. (1998). Carnosine modulates zinc and copper effects on amino acid receptors and synaptic transmission. *Neuroreport* 9(15)**,** 3503-3507.

Trombley, P.Q., and Shepherd, G.M. (1996). Differential modulation by zinc and copper of amino acid receptors from rat olfactory bulb neurons. *J Neurophysiol* 76(4)**,** 2536-2546.
