# Supplementary material for: Zinc as a Neuromodulator in the Central Nervous System with a Focus on the Olfactory Bulb
Source: Front Cell Neurosci. 2017 Sep 21;11:297. doi: 10.3389/fncel.2017.00297 (PMC5627021; doi:10.3389/fncel.2017.00297)
Supplement: Supplementary file 2 [file Table_2.docx]

Laura J. Blakemore and Paul Q. Trombley: “Zinc as a Neuromodulator in the Central Nervous System with a Focus on the Olfactory Bulb”

Table 2. Effects of zinc on voltage-gated ion channels expressed by olfactory bulb neurons.

| **Ion channel type** | **Effects of zinc** |
| --- | --- |
| Voltage-gated Ca2+ | 100 µM zinc inhibited HVA calcium-channel currents recorded from cultured rat OB neurons by approximately 60% (Horning and Trombley, 2001). |
| Voltage-gated Na+ | 100 µM zinc inhibited TTX-sensitive voltage-gated sodium currents recorded from cultured rat OB neurons by approximately 20% (Horning and Trombley, 2001). |
| Voltage-gated K+ | Application of zinc (10-300 µM) affected transient A-type currents, but not delayed rectifier-type currents, in PG cells in rat OB slices (Puopolo and Belluzzi, 1998). When the inactivation of A-type channels was removed with a hyperpolarizing step to -120 mV, zinc (100 µM and 300 µM) reduced the peak amplitude of the transient type A current in a concentration-dependent manner. However, when the membrane was depolarized starting from physiological holding potentials (e.g., -50 mV), zinc potentiated the A-type current (Puopolo and Belluzzi, 1998).  100 µM zinc inhibited delayed rectifier-type outward potassium currents recorded from cultured rat OB neurons at all voltages examined (Horning and Trombley, 2001).  Zinc (100 µM) inhibited A-type potassium currents recorded from cultured rat OB neurons when the membrane voltage was hyperpolarized but enhanced the current when the membrane was at or depolarized to a typical resting potential where most A-type channels are inactivated (Horning and Trombley, 2001). The effects of zinc on A-type currents are due to shifts in the voltage-dependence of steady-state activation and inactivation curves (Puopolo and Belluzzi, 1998). |

HVA: high-voltage activated; OB, olfactory bulb; PG: periglomerular; TTX: tetrodotoxin

**References**

Horning, M.S., and Trombley, P.Q. (2001). Zinc and copper influence excitability of rat olfactory bulb neurons by multiple mechanisms. *J Neurophysiol* 86(4)**,** 1652-1660.

Puopolo, M., and Belluzzi, O. (1998). Functional heterogeneity of periglomerular cells in the rat olfactory bulb. *Eur J Neurosci* 10(3)**,** 1073-1083.
